# Trauma‐focused guided self‐help interventions for posttraumatic stress disorder: A meta‐analysis of randomized controlled trials

**DOI:** 10.1002/da.23272

**Published:** 2022-05-27

**Authors:** Andy P. Siddaway, Richard Meiser‐Stedman, Verity Chester, Jack Finn, Cliodhna O. Leary, David Peck, Camilla Loveridge

**Affiliations:** ^1^ Institute of Health & Wellbeing, University of Glasgow Glasgow Scotland; ^2^ Department of Clinical Psychology Norwich Medical School, University of East Anglia, Norwich Research Park Norwich UK; ^3^ Department of Psychiatry Hertfordshire Partnership University NHS Foundation Trust, Little Plumstead Hospital Norwich UK

**Keywords:** cognitive‐behavioral, effectiveness, efficacy, posttraumatic stress disorder, review, self‐help, therapy

## Abstract

Trauma‐focused guided self‐help (TF‐GSH) is an important alternative to psychological therapy delivered by a therapist. This meta‐analysis evaluates the efficacy of TF‐GSH in reducing posttraumatic stress disorder  (PTSD) symptoms and comorbid depressive and anxiety symptoms. A total of 17 trials were included that compared a TF‐GSH intervention (*N* = 610) to various control comparators (*N* = 570). Control conditions included treatment as usual (*k* = 2), waiting list (*k* = 11), phone monitoring (*k* = 1), nontrauma writing (*k* = 1), general support (*k* = 1), and supportive counseling (*k* = 1). A moderate‐ to large‐sized effect favouring TF‐GSH was observed for PTSD (*k* = 17, *g* = −0.81, 95% confidence interval [CI]: −1.24, −0.39) and a moderate‐sized effect was observed for depressive (*k* = 13, *g* = −0.73, 95% CI: −1.16, −0.31) and anxiety (*k* = 11, *g* = −0.72, 95% CI: −1.18, −0.27) symptoms, with considerable heterogeneity. Moderator analyses were all not statistically significant. Results indicate that TF‐GSH is a promising treatment for PTSD and comorbid depressive and anxiety symptoms. We discuss the nature, extent, and quality of the literature to provide a point of departure for future research. TF‐GSH (and unguided self‐help) may not be appropriate for certain individuals at certain times. Exploring a broad range of treatment delivery modalities will move the field closer towards a model of evidence‐based care in which the likely appropriate dose and type of intervention can be matched to individuals based on presenting problems and other variables.

## 1 INTRODUCTION

Trauma exposure is near ubiquitous and posttraumatic stress disorder (PTSD) is a common psychological problem following trauma exposure (Koenen et al., 2017). A large body of research has tested the effectiveness and efficacy of pharmacological, psychological, and other treatments for PTSD. Meta‐analytic reviews indicate that some medications (fluoxetine, paroxetine, sertraline, venlafaxine and quetiapine) lead to a small reduction in PTSD symptom severity, (Hoskins et al., 2015, 2021) whereas a number of different trauma‐focused psychological therapies have demonstrated large effect sizes in reducing PTSD (Mavranezouli et al., 2020a, 2020b).

Although clinical guidelines for PTSD widely recommend trauma‐focused psychological therapy as the first‐line treatment, a number of barriers limit access to specialty mental health care in general and trauma‐focused psychological therapy in particular, including lack of confidence in treatment effectiveness; fear of increasing PTSD symptoms; perceived stigma associated with psychological therapy; practical barriers (e.g., transportation, limited treatment availability, especially in low‐ and middle‐income countries); and long waiting times (Koenen et al., 2017; Smith et al., 2020). Furthermore, not everyone benefits from trauma‐focused psychological therapy (around two‐thirds of individuals respond adequately (Bryant, 2019); therapy requires significant therapist input, is time consuming and costly (Hedman et al., 2011) to deliver, and places a high emotional demand on therapists; and many clinicians do not feel competent to deliver trauma‐focused psychological therapy (Finch et al., 2020).

In response to these barriers and the widespread need for treatment, there has been a growing interest in exploring alternative means of delivering trauma‐focused psychological therapies, including via supported and unsupported self‐help. Self‐help for PTSD that is delivered with active support and monitoring from a trained professional is known as trauma‐focused guided self‐help (TF‐GSH). TF‐GSH is a self‐administered intervention based on trauma‐focused cognitive behavioral therapy in which individuals are guided through written or electronic materials via face‐to‐face, email, internet, or phone call support. TF‐GSH is likely to be cheaper than psychological therapy that is delivered face‐to‐face by a therapist; requires less staff time, skill, training, and emotional involvement; and may be more accessible, convenient (e.g., fitting around work or school commitments), and appealing for some people, since it does not involve traveling to appointments in formal treatment settings.

A burgeoning literature has tested the efficacy of TF‐GSH for PTSD. The present meta‐analysis synthesizes the available randomized controlled trials on this topic with a view to obtaining an accurate estimate of the efficacy of TF‐GSH and providing a point of departure for future research in this area. Most of the trials included in the review also measured the impact of TF‐GSH for PTSD on depressive and anxiety symptoms because PTSD is often comorbid with other mental health problems (Horesh et al., 2017; O'Donnell et al., 2004). We, therefore, also present meta‐analyses of the efficacy of TF‐GSH for PTSD on depressive and anxiety symptoms.

## 2 METHODS

The meta‐analysis was conducted in accordance with best‐practice guidelines for conducting systematic reviews (Siddaway et al., 2019) and the Preferred Reporting Items for Systematic Reviews and Meta‐analyses standards (Moher, 2009). The systematic review protocol was preregistered with PROSPERO (CRD42015026026).

### 2.1 Inclusion and exclusion criteria

Randomized controlled trials that employed TF‐GSH and measured PTSD pre‐ and postintervention were potentially eligible for inclusion. To be included, TF‐GSH interventions were required to be delivered by a trained clinician on an individual (rather than group) basis, and to be trauma‐focused (i.e., incorporating an element of processing trauma memories and working with beliefs regarding the trauma[s]). To provide a comprehensive summary of the available evidence, explore heterogeneity, and increase the generalizability of the findings, no restrictions were applied for TF‐GSH medium (e.g., telephone, face to face), publication status, language, age group (children and adults), setting, or comparator group.

Studies were considered for inclusion where PTSD was the primary presenting difficulty. Data from studies that also measured changes in comorbid depressive and anxiety symptoms were included in additional meta‐analyses. PTSD status can potentially be determined by a qualified clinician's diagnosis, a standardized diagnostic interview, or a continuous self‐report measure of PTSD symptoms. Diagnostic and continuous approaches to measuring PTSD are both common in the literature and individuals with elevated PTSD symptoms experience significant functional impairment and are often referred for mental health services (Cohen, 2013). If a study included more than one PTSD outcome measure, a primary measure was selected based upon superiority of psychometric properties (i.e., published reliability and validity). If alternative measures had equivalent properties, we extracted data from clinician‐rated over self‐rated measures and/or the measure most frequently used in other included studies to attempt to reduce this potential source of heterogeneity. Total scores were used to calculate treatment effects where studies include both subscale and total scores. During the data extraction phase, to incorporate all relevant evidence within the review, a decision was made to derestrict the eligibility criteria to include studies where at least 70% of the sample reached clinical levels of PTSD symptoms as defined by a standardized PTSD outcome measure. This approach is consistent with best practice guidelines for conducting systematic reviews (Siddaway et al., 2019). No restrictions were placed on the type of trauma, the amount of time since the traumatic event, the chronicity of PTSD, or comorbid mental health problems. Studies were excluded if they were sampled from specific groups that would likely significantly affect the effectiveness of TF‐GSH or preclude suitability for psychological intervention (eg, personality disorder, neurodevelopmental disorder, learning disability, severe depression, or substance dependency).

### 2.2 Literature search

Comprehensive search strategies were developed by combining key and index terms covering PTSD and TF‐GSH, with a comprehensive range of search terms within each concept (see Supporting Information Material). Six electronic databases (PubMed, Medline, Embase, PsycINFO, Web of Science, PILOTS) were searched from 1980 (when PTSD was introduced) to January 2021. The reference lists of included articles and relevant review articles were hand‐searched, and unpublished dissertations and theses were sought via ProQuest Dissertation & Theses and OpenGrey to minimize publication bias. The Cochrane Library, British Medical Journal Best Practice, and the NICE Evidence Search engine were also searched. Each database was searched separately. Ongoing studies were identified by searching across a range of trials registers (via the International Clinical Trials Registry Platform Search Portal) and information requests were emailed to the principal and key authors of included studies to identify any additional unpublished or published studies that may be relevant for inclusion.

All identified studies were exported to EndNote X9 for Windows, where duplicates were removed. To determine study eligibility, all titles and abstracts were screened independently by two researchers (C.L. and A.P.S.), who also conducted the second full‐text screening independently. Disagreements or uncertainties were discussed with the senior researcher supervising the project (R.M‐S.).

### 2.3 Data analysis

Cohen's *d* was computed to represent the between‐group treatment effect for each study by subtracting the mean postintervention score of the control group from the mean postintervention score of the experimental group and dividing the result by the pooled standard deviation. Cohen's *d* was transformed into Hedge's *g* to reduce the bias inherent in *d* when *N* is small. An effect size of 0.8 was considered large, 0.5 moderate, and 0.2 small (Cohen, 2013). Study authors were contacted for additional information when an effect size or data to compute an effect size were not reported.

Separate random effects meta‐analyses were conducted for PTSD, depression, and anxiety problem effect sizes using R Version 4.1, 2021 (R Core Team, 2014). Heterogeneity was assessed using the Q statistic, τ^*2*^, and *I* ^*2*^. τ^*2*^ estimates the amount of total heterogeneity and is measured on the same scale as the effect size itself (in this meta‐analysis: *g*). *I* ^*2*^ is the percentage of the total variability that is due to true (i.e., between‐study) heterogeneity rather than sampling error. Percentages of around 25% (*I* ^*2*^ = 25), 50% (*I* ^*2*^ = 50), and 75% (*I* ^*2*^ = 75) indicate low, medium, and high heterogeneity, respectively (Higgins & Thompson, 2002). Subsequently, several substantive and methodological moderators were examined in relation to PTSD effect sizes. Small *k*s and missing values (see Tables 1 and 2) precluded the exploration of any further moderators and categorical moderators were only examined when there were at least six effect sizes per subgroup (Borenstein et al., 2009).

**Table 1:** Characteristics of included studies: Methods and participants

Author, year	Year (s) conducted	Country	Population *N*	Diagnosis/symptoms (outcome)	% PTSD at entry	Trauma	Combat	Age (*M*, [*SD*], range)	Sex (% Female)	Education level (% university degree/tertiary)
Acosta (2017)	NR	USA	War Veterans, 162	CAPS	79 (Dx)	War	Combat	34, (8.1), 22–64	7	39%
Engel et al. (2015)	NR	USA	War Veterans, 80	PCL	100 (Dx)	War	Combat	36.4, (8.7), NR	18.6%	NR
Gawlytta et al. (2017)	NR	Germany	Medical, 34	PCL	73.5 (Dx)	Sepsis	Noncombat	55, (NR), 47–62	48%	34
Ivarsson et al. (2014)	2012	Sweden	GP, 62	CAPS	100 (Dx)	Diverse	Noncombat	46, (11.7), 21–67	82.3	56.5
Knaevelsrud and Maercker (2009)	2003‐2004	Switzerland	GP, 96	IES‐R (A, I)	100 (CO)	Diverse	Noncombat	35, 18–68	90	44
Knaevelsrud et al. (2015)	2009‐2011	Germany (Iraqis/Arab‐speakers)	GP, 159	PDS	100 (CO)	War, terror	Combat	28.1, (7.43), 18–56	72	75
Knaevelsrud et al. (2017)	2008 – 2012	Germany	War Veterans, 94	PDS	100 (CO)	War	Combat	71.4, 4.7, 63–85	64.9	NR
Lange et al. (2003)	NR	Netherlands	GP, 184	IES (A, I)	90 (CO)	Diverse	Noncombat	NR	NR	NR
Latif et al. (2021)	2018 – 2019	Pakistan	GP, 50	IES‐R	100 (Dx)	Diverse	Noncombat	26.85, 4.1, NR	100	NR
Lehavot et al. (2021)	NR	USA	Veterans, 102	PCL	100 (Dx)	War	Combat	NR	100	NR
Lewis et al. (2017)	2013‐2014	UK	GP, 42	CAPS	100 (CO)	Diverse	Noncombat	39.29, 12.7, 20–65	59.5	85.7
Litz et al. (2007)	Ended 2005	USA	GP, service members, 43	PSS‐I	100 (Dx)	Combat, terror	Combat	39.25, (12.2)	22	NR
Nieminen et al. (2016)	NR	Sweden	GP, 56	TES	100 (Dx)	Childbirth	Noncombat	34.6, (4.8), 25–50	100	80.4
Sloan et al. (2012)	2009‐2010	USA	GP, 46	CAPS	100 (Dx)	MVA	Noncombat	40.65, (13.1)	65	41
Spence et al. (2011)	2010	Australia	GP, 44	MINI‐PTSD	100 (Dx)	Diverse	Noncombat	42.6, (13.1), 21–68	81	52
Vinke et al. (2019)	NR	Netherlands	GP/students, 112	PDS	100 (CO)	Diverse	Noncombat	23.33 (5.16), NR	73.9	NR
Wagner et al. (2006)	NR	Switzerland	GP, 55	IES (A, I)	100 (Dx)	Loss	Noncombat	37.0, (10.2), 19–68	93	31

*Note*: Age and sex of total study participants. A = PTSD avoidance subscale; CAPS = Clinician‐Administered PTSD Scale (Blake et al., 1990); GP = general population; I = PTSD intrusion subscale; IES = Impact of Event Scale (Horowitz et al., 1979; IES Dutch version, Brom & Kleber, 1985); IES‐R = Impact of Event Scale‐Revised (Weiss & Marmar, 1997); MINI‐PTSD = PTSD module of the Mini‐International Neuropsychiatric Interview (Sheehan et al., 1998); MVA = Motor Vehicle Accident; NR = not reported; PDS = Posttraumatic Diagnostic Scale (Foa et al., 1997); PSS‐I = PTSD Symptom Scale‐Interview (Foa & Tolin, 2000).

Abbreviation: PTSD, posttraumatic stress disorder.

**Table 2:** Characteristics of included studies: Treatment modality and duration

Author (year)	Intervention, *N*	Control group, *N*	Mode of intervention	Length of intervention (Weeks)	Hours of treatment received (hours, *M*, [*SD*])	Proportion who completed treatment (%)	Format of therapist input
Acosta (2017)	24 modules (12 core modules), 81	TAU, 81	Internet	12	Client: 8 Therapist: NR	38.3	Telephone call, text message, Face‐to‐face
Engel et al. (2015)	DESTRESS, 43	TAU, 37	Internet	6	NR	35	Internet
Gawlytta et al. (2017)	Trauma writing, 16	WL, 18	Internet	5	Client: 8.3 h Therapist: NR	NR	Internet
Ivarsson et al. (2014)	8 CBT modules, 31	General support, 31	Internet	8	NR	39	Email
Knaevelsrud and Maercker (2009)	Interapy, 49	WL, 47	Internet	5	Client: 7.5 Therapist: 7.5	NR	NR
Knaevelsrud et al. (2015)	Interapy, 79	WL, 80	Internet	5	Client: 7.5 Therapist: 5.8	100	Email, Telephone call
Knaevelsrud et al. (2017)	Trauma writing, 47	WL, 47	Internet	6	Client: 8.25 Therapist: 0.75‐0.83	87.2	Email
Lange et al. (2003)	Interapy, 69	WL, 32	Internet	5	Client: 7.5 Therapist: NR	63.9	Internet
Latif et al. (2021)	Culturally informed CBT GSH, 25	WL, 25	Internet	12	Client: NR Therapist: 2.6	100	Face‐to‐face
Lehavot et al. (2021)	DESTRESS, 51	Phone monitoring, 51	Internet	8	Client: NR Therapist: 2.25	76	Telephone call
Lewis et al. (2017)	8 online modules, 21	WL, 21	Internet	8	Client: NR Therapist: 2.5	35.7	Telephone call, Face‐to‐face
Litz et al. (2007)	DESTRESS, 14	Supportive counseling, 17	Internet	8	NR	NR	Face‐to‐face, Email, Telephone call,
Nieminen et al. (2016)	Trauma writing, 28	WL, 28	Internet	8	NR	54	Email
Sloan et al. (2012)	Modified WET, 22	WL, 24	Written handouts	5	Client: 3.7 Therapist: 1.1	91	Face‐to‐face
Spence et al. (2011)	7 CBT lessons, 23	WL, 19	Internet	8	NR	78	Email, Telephone call
Vinke et al. (2019)	Single session WET	Nontrauma writing	Internet	1	Client: .75 Therapist: NR	38	Internet
Wagner et al. (2006)	Interapy, 26	WL, 25	Internet	5	Client: 7.5 Therapist: NR	NR	Email

*Note*: M, mean; *N*, sample size; NR, not reported; *SD*, standard deviation.

Abbreviations: TAU, treatment as usual; WET, writing exposure therapy; WL, waiting list.

### 2.4 Risk of bias

The methodological quality of included studies was determined using the Cochrane Collaboration's Risk‐of‐Bias Tool (Higgins & Green, 2011). Assessments are made of the following 7 potential sources of bias: (1) random sequence generation (selection bias), (2) allocation concealment (selection bias), (3) blinding of participants and personnel (performance bias), (4) blinding of outcome assessment (detection bias), (5) incomplete outcome data (attrition bias), (6) selective reporting (reporting bias), and (7) other sources of bias. The risk of bias for each domain was scored as low (0), high (2), or unclear (1). The risk of bias assessment was performed by C. L and J. F. with R.M‐S consulted in case of uncertainty.

Three strategies were used to assess publication bias. First, a funnel plot was created to visually search for evidence of bias, which would be apparent in an asymmetrical plot. Next, asymmetry was assessed using Egger's weighted regression test (Egger et al., 1997) and Duval and Tweedie's trim and fill method (Duval & Tweedie, 2000).

## 3 RESULTS

### 3.1 Study characteristics

**Figure 1:**
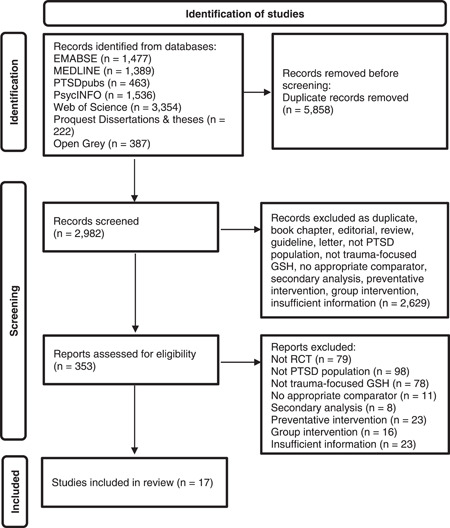
Preferred reporting items for systematic reviews and meta‐analysis flow chart of study selection process

A flow diagram of study identification and selection is presented in Figure 1. The characteristics of included studies are described in Engel et al., 2015; Gawlytta et al., 2017; Ivarsson et al., 2014; Knaevelsrud & Maercker, 2009; Knaevelsrud et al., 2015, 2017; Lange et al., 2003; Latif et al., 2021; Lehavot et al., 2021; Lewis et al., 2017; Litz et al., 2007; Nieminen et al., 2016; Sloan et al., 2012; Spence et al., 2011; Tables 1 and 2 (Acosta, 2017; Vinke et al., 2019; Wagner et al., 2006). The review identified 17 trials comparing a TF‐GSH intervention (total *N* = 610) with a control group comparator (total *N* = 570) for individuals experiencing PTSD. Control conditions included treatment as usual (*k* = 2), (Acosta, 2017; Engel et al., 2015) waiting list (*k* = 11), (Gawlytta et al., 2017; Knaevelsrud & Maercker, 2009; Knaevelsrud et al., 2015, 2017; Lange et al., 2003; Latif et al., 2021; Lewis et al., 2017; Nieminen et al., 2016; Sloan et al., 2012; Spence et al., 2011; Wagner et al., 2006) phone monitoring (*k* = 1), (Lehavot et al., 2021) nontrauma writing (*k* = 1), (Vinke et al., 2019) general support (*k* = 1), (Ivarsson et al., 2014) and supportive counseling (*k* = 1) (Litz et al., 2007). Trials evaluating the efficacy of TF‐GSH were conducted in a range of countries and in relation to a broad range of traumas. All samples involved adults. All studies were published and reported in English (Morrison et al., 2012) and used manualised treatments. All interventions included evidence‐based principles of trauma‐focused psychological therapy such as written exposure exercises (i.e. imaginal exposure) and psychoeducation about PTSD symptoms and the mechanisms of treatment strategies. Several different TF‐GSH protocols were tested. Therapist input entailed less than 1 h (*k* = 1), (Vinke et al., 2019) 3.7–4.5 h (*k* = 4), (Gawlytta et al., 2017; Lange et al., 2003; Latif et al., 2021; Sloan et al., 2012) 7‐5‐8.3 h (*k* = 5), (Knaevelsrud & Maercker, 2009; Knaevelsrud et al., 2015, 2017; Lehavot et al., 2021; Wagner et al., 2006) and 23.6 h (Litz et al., 2007) guided support. One (Sloan et al., 2012) of the included studies used written materials as opposed to being delivered via the internet. TF‐GSH generally involved less than 8 h intervention and therapist support was delivered via the internet (*k* = 5), (Acosta, 2017; Engel et al., 2015; Gawlytta et al., 2017; Lehavot et al., 2021; Vinke et al., 2019) email (*k* = 3), (Ivarsson et al., 2014; Nieminen et al., 2016; Wagner et al., 2006) phone (*k* = 2), (Latif et al., 2021; Lewis et al., 2017) face to face (*k* = 2), (Lange et al., 2003; Sloan et al., 2012) or through combinations of these (*k* = 3) (Knaevelsrud et al., 2015; Litz et al., 2007; Spence et al., 2011). Dropout rates ranged from 0 (Knaevelsrud et al., 2015; Lange et al., 2003) to 65%, (Engel et al., 2015; Latif et al., 2021) with an average of 36% (based on *k* = 12). 6 of the 17 included studies used a clinician‐rated measure of PTSD.

### 3.2 Meta‐analyses

**Figure 2:**
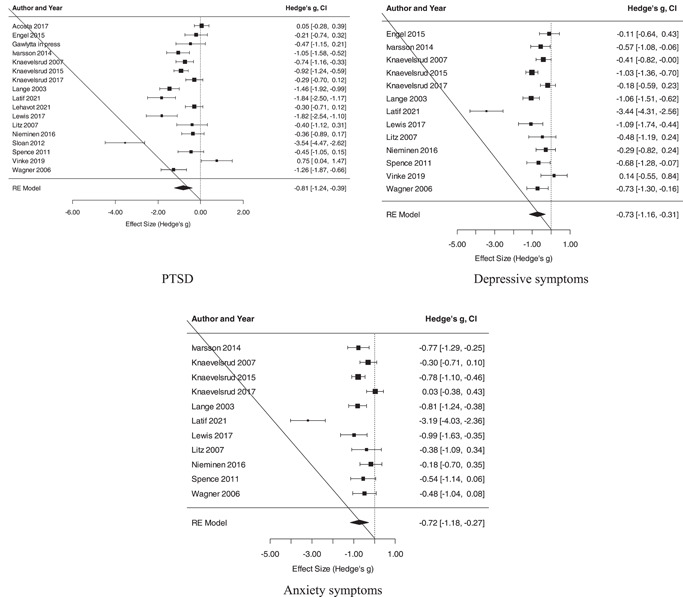
Forest plots of effect sizes for PTSD, depressive symptoms, and anxiety symptoms. PTSD, posttraumatic stress disorder

Three forest plots summarize the PTSD, depressive symptoms, and anxiety symptoms effect sizes (Figure 2). A large effect favouring TF‐GSH was observed for PTSD (*k* = 17, *g* = −0.81, 95% confidence interval [CI]: −1.24, −0.39), with considerable heterogeneity among effect sizes (*Q*(16) = 122.60, *p* < .001, τ^*2*^ = 0.70, *I* ^*2*^ = 91%). A moderate to large effect favouring TF‐GSH was observed for depressive symptoms (*k* = 13, *g* = −0.73, 95% CI: −1.16, −0.31), with considerable heterogeneity among effect sizes (*Q*(12) = 66.96, *p* < .001, τ^*2*^ = 0.53, *I* ^*2*^ = 89%); a similar‐sized effect was observed for anxiety symptoms (*k* = 11, *g* = −0.72, 95% CI: −1.18, −0.27), with considerable heterogeneity among effect sizes (*Q*(10) = 55.23, *p* < .001, τ^*2*^ = 0.50, *I* ^*2*^ = 89%).

### 3.3 Moderator analyses and risk of bias

Several outliers (Latif et al., 2021; Sloan et al., 2012) were identified (see Figure 2). See Figure S2 for funnel plots of effect sizes for PTSD, depressive symptoms, and anxiety symptoms with these outliers removed. Removing the Sloan et al. (2012) PTSD effect size reduced the strength of the effect of TF‐GSH on PTSD by *g* = 0.15 and halved the degree of heterogeneity among PTSD effect sizes (reducing τ^*2*^ by 0.35). A moderate to large effect favouring TF‐GSH was observed (*k* = 16, *g* = −0.66, 95% CI: −0.99, −0.34), with some heterogeneity among effect sizes (*Q*(15) = 84.92, *p* < .001, τ^*2*^ = 0.35, *I* ^*2*^ = 85%).

Removing the Latif et al (Latif et al., 2021) depressive symptoms effect size reduced the strength of the effect of TF‐GSH on depressive symptoms by *g* = 0.17 and almost eliminated heterogeneity among depressive symptom effect sizes (reducing τ^*2*^ by 0.44). A moderate effect favouring TF‐GSH was observed for depressive symptoms (*k* = 12, *g* = −0.56, 95% CI: −0.78, −0.33), with a small degree of heterogeneity among effect sizes (*Q*(11) = 27.14, *p* < .01, τ^*2*^ = 0.09, *I* ^*2*^ = 58%). Removing the Latif et al (Latif et al., 2021) anxiety symptoms effect size reduced the strength of the effect of TF‐GSH on anxiety symptoms by *g* = 0.21 and almost eliminated heterogeneity among anxiety symptom effect sizes (reducing τ^*2*^ by 0.45). A moderate effect favouring TF‐GSH was observed for anxiety symptoms (*k* = 10, *g* = −0.51, 95% CI: −0.78, −0.33), with a small degree of nonsignificant heterogeneity among effect sizes (*Q*(9) = 17.05, *p* = .04, τ^*2*^ = 0.05, *I* ^*2*^ = 48%).

There was little to no evidence of publication bias (see Supplementary material). Risk of bias total score (*Q*(1) = 0.05, *p* = .82), dropout rate (*Q*(1) = 2.18, *p* = .14), length of intervention (*b* = −.04, *p* = .62), clinician versus self‐rated measure of PTSD (*Q*(1) = 1.11, *p* = .29), hours of therapist contact (*b* = .12, *p* = .73), combat trauma versus other trauma (*Q*(1) = 3.10, *p* = .08), active vs. waiting list control condition (*Q*(1) = 1.66, *p* = .20), mean age (*b* = .00, *p* = .99), and sample gender composition (*b* = −.01, *p* = .36) did not significantly moderate PTSD effect sizes. As type of trauma and control condition each approached statistical significance as moderating variables, we note that TF‐GSH appeared to be much less effective (but not statistically significantly different) for combat (*g* = −0.35, 95% CI: −0.65, −0.05) versus noncombat (*g* = −1.09, 95% CI: −1.71, −0.48) groups, and when compared to more active control conditions (*g* = −0.44, 95% CI: −1.13, 0.25) versus waiting list control conditions (*g* = −1.01, 95% CI: −1.53, −0.49).

We conducted several sensitivity analyses to further investigate the robustness of our moderator results. Arguably, criteria (3) of the Cochrane Collaboration's Risk‐of‐Bias Tool (blinding of participants and personnel) is of limited relevance to a psychological intervention where participants could not be blinded to the allocation they had received; however, re‐running the moderator analysis excluding this item did not render risk of bias a statistically significant moderator of PTSD effect sizes (*Q*(1) = 0.05, *p* = .81).

Control conditions are conceptualized differently in the literature (e.g., treatment as usual is considered to be equivalent to waiting list/no treatment in some trials and clinical guidelines). It is possible that our active versus waiting list control condition moderator analysis concealed potential group differences. We therefore conducted a moderator analysis comparing trials that used an “active” control condition (supportive counseling, phone monitoring, non‐trauma writing, general support; *k* = 4, *g* = −0.63, 95% CI: −1.69, 0.42, *I* ^*2*^ = 90.1%) to trials that used a “passive” control condition (waiting list, treatment as usual; *k* = 13, *g* = −0.86, 95% CI: −1.33, −0.39, *I* ^*2*^ = 92.0%). This revealed a nonsignificant moderator effect (*Q*(1) = 0.18, *p* = .67).

Finally, as studies varied widely in how they reported duration of TF‐GSH, we examined whether excluding two studies (Knaevelsrud & Maercker, 2009; Knaevelsrud et al., 2015) that delivered the Interapy intervention, which involved a high degree of therapist input, impacted the PTSD results. We found a large effect favouring TF‐GSH for PTSD (*k* = 15, *g* = −0.82, 95% CI: 0.00, −1.31), with considerable heterogeneity among effect sizes (*Q*(14) = 119.63, *p* < .001, τ^*2*^ = 0.84, *I* ^*2*^ = 92%). This result is almost identical (differing by *g* = 0.01) to our original result.

## 4 DISCUSSION

We conducted a meta‐analysis to examine the efficacy of TF‐GSH for PTSD. Secondary analyses also examined the effect of TF‐GSH on depressive symptoms and anxiety symptoms. The review identified 17 trials comparing a TF‐GSH intervention (total *N* = 610) with a control group comparator (total *N* = 570) for individuals experiencing PTSD. TF‐GSH interventions were designed to reduce symptoms of PTSD and comprised elements such as written exposure exercises (i.e., imaginal exposure) and psychoeducation about PTSD.

A statistically significant, moderate to large‐sized (Cohen, 2013) effect favouring TF‐GSH was observed for PTSD and a statistically significant, moderate‐sized (Cohen, 2013) effect was observed for depressive and anxiety symptoms, providing evidence of the potential value of TF‐GSH for PTSD and comorbid mental health problems. There was no evidence of publication bias, suggesting that the meta‐analytic results are unlikely to have been artificially inflated by file‐drawer effects. There was also no evidence that methodological rigour affected the strength of effects, furthering underpinning the robustness of the findings. Treatment benefits were demonstrated across severities of PTSD, suggesting that the effects documented here are not limited to “mild” PTSD presentations. That an intervention involving relatively little clinician input can achieve substantial change is consistent with the well‐established finding that most people, irrespective of trauma type and exposure, are able to find a way to process and adjust to potentially traumatic experiences without the need for professional involvement (Ehlers & Clark, 2000).

The effect sizes observed here for TF‐GSH are similar in magnitude to those observed for internet‐delivered cognitive behavioral therapy (iCBT) for PTSD (Kuester et al., 2016; Sijbrandij et al., 2016) and telehealth interventions for PTSD (Sloan et al., 2011). In contrast to the current review, the meta‐analyses of iCBT and telehealth interventions included relatively few studies that used a clinical sample. The present review only included one study that would not be classed as an iCBT and hence might be considered an update or extension of the meta‐analyses of iCBT, albeit restricted to iCBT interventions where therapist support was available. While iCBT may have advantages for many populations in terms of accessibility, there is a paucity of research on therapy delivered through low technology means (e.g., printed materials). In some contexts and for some people (e.g., those who are digitally excluded), it may be that simpler, noninternet‐delivered TF‐GSH interventions are more accessible, easily implemented, and/or disseminated, and potentially cheaper.

A considerable degree of heterogeneity was observed, especially among PTSD effect sizes, potentially suggesting that caution should be applied when interpreting the summary effects, (Deeks et al., 2011) One outlier (demonstrating a much stronger effect of TF‐GSH) was identified in each meta‐analysis (Latif et al., 2021; Sloan et al., 2012). Removing the three identified outliers somewhat reduced the strength of associations, substantially reduced the degree of heterogeneity among PTSD effect sizes, and almost eliminated heterogeneity among depressive symptom and anxiety symptom effect sizes. The results with outliers removed are probably the most accurate estimate of the effect of TF‐GSH. The small number of included studies means that outliers are not clearly attributable to systematically different methodological features or biases when compared to the other studies included in the meta‐analysis and potential reasons for the three outlying effect sizes can only be very tentatively speculated upon (e.g., use of written material [Sloan et al., 2012], study conducted in Pakistan [Latif et al., 2021]).

Moderator analyses were conducted in an attempt to explain the observed heterogeneity; all were not statistically significant. To inform future research, we reported that TF‐GSH appeared to be much less effective (but not statistically significantly different) for combat versus noncombat groups and when compared to more active control conditions versus waiting list control conditions. Given the potential for low statistical power because of small numbers of effect sizes and the small sample sizes of included trials, (Hunter & Schmidt, 2004) we note that failure to obtain a statistically significant difference among subgroups was not interpreted as evidence that effect sizes are the same across subgroups (Borenstein et al., 2009).

The average dropout rate from TF‐GSH for the studies included in this review is 36% (based on *k* = 12). This figure is somewhat higher than that found in therapist‐administered individual trauma‐focused psychological therapies, although we note that a moderator analysis found that dropout rate did not appear to influence outcomes (Lewis et al., 2020). Some of the included studies outlined reasons for dropouts. For example, one study (Lewis et al., 2017) reported that the majority of dropout occurred before participants began TF‐GSH, indicating a reluctance to engage in GSH as opposed to an issue related to the tolerability of the treatment itself.

Although the present meta‐analytic results suggest that TF‐GSH appears to be a promising potential intervention for PTSD, several limitations are apparent in the available literature, each of which point to future research directions for TF‐GSH trials. The included studies generally involved adult samples, although a small number of studies included older adults. To our knowledge, no study has been conducted to test the efficacy of TF‐GSH in children or adolescents, though our research group is in the early stages of conducting such a study. Female participants were also over‐represented, which may be consistent with the higher prevalence of PTSD in this group. Many TF‐GSH trials excluded participants with psychiatric comorbidities such as severe depression, psychosis, substance abuse, and dissociative symptoms. This review can only, therefore, provide a preliminary indication regarding whether individuals with significant comorbidities may benefit from TF‐GSH.

A further key issue to consider in future research is the degree and manner of therapist involvement because TF‐GSH is intended to be less intensive and cheaper than therapist‐delivered psychological therapy. The studies included in this meta‐analysis employed self‐help with some clinician guidance (i.e. the interventions were not psychological therapy).

The trials involved widely differing amounts of therapist contact. Some trials involved a relatively high degree of therapist contact, which obviously brings into question whether therapist time would have been more effectively used delivering traditional individual face‐to‐face psychological therapy. However, we note that moderator and sensitivity analyses both supported the robustness of our findings and demonstrated that hours of therapist contact did not appear to influence outcomes and TF‐GSH is still efficacious even when the most therapist‐heavy interventions are excluded from analyses. Like trauma‐focused psychological therapy, TF‐GSH (and unguided self‐help) may not be appropriate for certain individuals at certain times. Based on the information reported, it is not clear whether any adverse effects occurred in the studies included in this meta‐analysis. The safety and suitability of different forms of therapist contact and interventions warrant further investigation with respect to adverse events, access to emergency care, and so on.

Further trials are also needed to elucidate whether particular types of TF‐GSH are most effective (e.g., comparing written materials with the much more common internet‐based materials), and whether TF‐GSH is more effective for different populations, trauma types, and individuals meeting criteria for PTSD versus complex PTSD. One possibility raised here which requires further research attention is that TF‐GSH may be less effective for individuals who have experienced combat. Although likely contradictions could be speculated upon (e.g., individuals who are experiencing dissociative symptoms or who are at high risk to themselves or others), ultimately, what works for whom and under what circumstances is an empirical question. Our meta‐analysis highlighted that the majority of the existing trials used a waiting list or treatment as usual control condition. Adequately powered non‐inferiority trials comparing TF‐GSH to in‐person therapist‐led treatment (the standard delivery of individual TF‐CBT) in clinical samples, as well as cost‐effectiveness studies and implementation studies, are much needed to help inform the future planning of services for people with PTSD.

## 5 CONCLUSION

Trials examining the efficacy of GSH interventions for a range of psychological problems are proliferating. The present meta‐analysis found that TF‐GSH has a moderate to large‐sized impact on PTSD and a moderate‐sized impact on depressive and anxiety symptoms. GSH is likely to be cheaper than psychological therapy that is delivered face‐to‐face to individuals by a therapist; requires less staff time, skill, training, and emotional involvement; and may be more accessible, convenient (e.g., fitting around work or school commitments), and appealing for some people, since it does not involve traveling to appointments in formal treatment settings.

The continued proliferation of research into alternative treatments for PTSD is welcome, as doing so is only likely to increase access to evidence‐based interventions and help to continue to refine interventions. Exploring a broad range of treatment delivery modalities will help the field to move closer and closer towards a model of evidence‐based care in which the likely appropriate dose and type of intervention can be matched to individuals based on their presenting problems and other variables.

### PEER REVIEW

The peer review history for this article is available at https://publons.com/publon/10.1002/da.23272

## Supporting information

Supporting information.

## Data Availability

The data that support the findings of this study are available from the corresponding author upon reasonable request.
