# Supplementary material for: Trauma‐focused guided self‐help interventions for posttraumatic stress disorder: A meta‐analysis of randomized controlled trials
Source: Depress Anxiety. 2022 May 27;39(10-11):675–85. doi: 10.1002/da.23272 (PMC9796235; doi:10.1002/da.23272)
Supplement: Supplementary file 1 — Supporting information. [file DA-39-675-s001.docx]

Table S1. PRISMA-P (Preferred Reporting Items for Systematic review and Meta-Analysis Protocols) 2015 checklist

| Section and topic | Item no. | Checklist item |
| --- | --- | --- |
| Administrative information | | |
| Title: Update | 1a | The manuscript is identified as a systematic review and meta-analysis |
| Title: Identification | 1b | The meta-analysis is described as the first review of its kind |
| Registration | 2 | The name of the registry the review was registered with is reported |
| Authors: Contact | 3a | Author contact details are provided; the mailing address of the corresponding author is also provided |
| Authors: Contributions | 3b | Author contributions are described in the manuscript |
| Amendments | 4 | The review does not represent an amendment of a previously completed or published protocol |
| Support: Sources | 5a | Sources of financial or other support for the review are listed |
| Support: Sponsor | 5b | There was no review funder or sponsor |
| Support: Role of sponsor or funder | 5c | The review was not funded |
| Introduction | | |
| Rationale | 6 | The rationale for the review is provided in the context of what is already known |
| Objectives | 7 | An explicit statement of the question(s) the review will address is included |
| Methods | | |
| Eligibility criteria | 8 | The study characteristics and report characteristics to be used as criteria for eligibility for the review are described |
| Information sources | 9 | All information sources are described in full, with dates of coverage |
| Search strategy | 10 | The search strategy is described in detail in the manuscript and supplement |
| Study records: Data management | 11a | The mechanism(s) used to manage records and data are reported |
| Study records: Selection process | 11b | The process of study selection is described |
| Study records: Data collection process | 11c | The method of extracting data from reports is described |
| Data items | 12 | Variables for which data were sought are described |
| Outcomes and prioritization | 13 | All outcomes for which data were sought are described |
| Risk of bias in individual studies | 14 | Methods for assessing risk of bias of individual studies is described, as is how this information was examined as a moderator variable |
| Data synthesis | 15a | Criteria under which study data were quantitatively synthesized are described |
|  | 15b | Methods of combining data from studies and exploring consistency are described |
|  | 15c | Additional sensitivity analyses are described |
|  | 15d | Quantitative synthesis was appropriate |
| Meta-bias(es) | 16 | Assessment of meta-bias(es) are described |
| Confidence in cumulative evidence | 17 | The manuscript describes how the strength of the body of evidence was assessed |

###### Search Strategies

**EMBASE**

1. posttraumatic stress OR posttraumatic stress disorder OR “posttraumatic stress disorder (PTSD)” [EMTREE Terms: major descriptors]

2. (“posttraumatic stress disorder” OR “post-traumatic stress disorder” OR “PTSD” OR “post traumatic stress disorder” OR “posttraumatic” OR “post-traumatic” OR “traumatic event”) [Title, Abstract]

3. self-help OR self care [EMTREE Terms: major descriptors]

4. (“web*” OR “comput*” OR “internet” OR “online” OR “bibliotherapy” OR “videotape” OR “audiotape” OR “etherapy” OR “cybertherapy” OR “e-health” OR “videoconferenc*” OR “videoteleconferenc*” “interapy” OR “tele*” OR “electronic” OR “skype” OR “instant messaging” OR “mobile” OR “tape” OR “DVD*” OR “CD*” OR “self-help” OR “self-care” OR “self-directed” OR “self-change” OR “self-management” OR “self-administ*” OR “guided self-help” OR “guided self-change” OR “guided” OR “self-exposure” OR “minimal contact” OR “minimal therapist contact” OR “reduced contact” OR “reduced therapist contact” OR “limited contact” OR “limited therapist contact” OR “therapist assisted”) [Title, Abstract]

5. crossover procedure OR double blind procedure OR randomized controlled trial OR single blind procedure OR controlled clinical trial [EMTREE Terms: not ‘exploded’]

6. (“random*” OR “factorial” OR “crossover*” OR “cross over*” OR “cross-over” OR “placebo” OR (doubl* blind*) OR (singl* blind*) OR “assign*” OR “allocate*” OR “volunteer*”) [Title, Abstract]

7. 1 OR 2

8. 3 OR 4

9. 5 OR 6

10. 7 AND 8 AND 9 [Limit to: Publication Year 1980-2015]

**MEDLINE**

1. stress disorders, post-traumatic/ OR stress disorders, traumatic/ OR combat disorders/ [MeSH Terms: major descriptors]

2. (“posttraumatic stress disorder” OR “post-traumatic stress disorder” OR “PTSD” OR “post traumatic stress disorder” OR “posttraumatic” OR “post-traumatic” OR “traumatic event”) [Title, Abstract]

3.bibliotherapy OR telemedicine [MeSH Terms: major descriptors]

4. (“web*” OR “comput*” OR “internet” OR “online” OR “bibliotherapy” OR “videotape” OR “audiotape” OR “etherapy” OR “cybertherapy” OR “e-health” OR “videoconferenc*” OR “videoteleconferenc*” “interapy” OR “tele*” OR “electronic” OR “skype” OR “instant messaging” OR “mobile” OR “tape” OR “DVD*” OR “CD*” OR “self-help” OR “self-care” OR “self-directed” OR “self-change” OR “self-management” OR “self-administ*” OR “guided self-help” OR “guided self-change” OR “guided” OR “self-exposure” OR “minimal contact” OR “minimal therapist contact” OR “reduced contact” OR “reduced therapist contact” OR “limited contact” OR “limited therapist contact” OR “therapist assisted”) [Title, Abstract]

5. clinical trials as topic [MeSH Term: not ‘exploded’]

6. (“random*’ OR “placebo” OR “trial” OR “group*”) [Title, Abstract]

7. controlled clinical trial or randomized controlled trial [Publication Type]

8. 1 OR 2

9. 3 OR 4

10. 5 OR 6

11. 8 AND 9 AND 10

12. 11 [Limit to: Publication Year 1980-2015]

**PsycINFO**

1. posttraumatic stress disorder [Subject Term: major descriptor]

2. (“posttraumatic stress disorder” OR “post-traumatic stress disorder” OR “PTSD” OR “post traumatic stress disorder” OR “posttraumatic” OR “post-traumatic” OR “traumatic event”) [Title, Abstract]

3. self management OR self help techniques [Subject Terms: major descriptors]

4. (“web*” OR “comput*” OR “internet” OR “online” OR “bibliotherapy” OR “videotape” OR “audiotape” OR “etherapy” OR “cybertherapy” OR “e-health” OR “videoconferenc*” OR “videoteleconferenc*” “interapy” OR “tele*” OR “electronic” OR “skype” OR “instant messaging” OR “mobile” OR “tape” OR “DVD*” OR “CD*” OR “self-help” OR “self-care” OR “self-directed” OR “self-change” OR “self-management” OR “self-administ*” OR “guided self-help” OR “guided self-change” OR “guided” OR “self-exposure” OR “minimal contact” OR “minimal therapist contact” OR “reduced contact” OR “reduced therapist contact” OR “limited contact” OR “limited therapist contact” OR “therapist assisted”) [Title, Abstract]

5. clinical trials [Subject Term: not ‘exploded’]

6. (“random*” OR “factorial” OR “crossover*” OR “cross over*” OR “cross-over” OR “placebo” OR (doubl* blind*) OR (singl* blind*) OR “assign*” OR “allocate*” OR “volunteer*” OR “control*” OR (clinical trial)) [Title, Abstract]

7. 1 OR 2

8. 3 OR 4

9. 5 OR 6

10. 7 AND 8 AND 9

**PILOTS**

1. (“self help techniques” OR “computer assisted psychotherapy” OR “bibliotherapy” OR “telemedicine”) [Subject Terms: exact]

2. “clinical trial” OR “randomized clinical trial” [Subject Terms: exploded]

3. 1 AND 2 [Limit to: Publication Year 1980-2015]

**Web of Science**

1. (“posttraumatic stress disorder” OR “post-traumatic stress disorder” OR “PTSD” OR “post traumatic stress disorder” OR “posttraumatic” OR “post-traumatic” OR “traumatic event”) [Topic]
2. (“web*” OR “comput*” OR “internet” OR “online” OR “bibliotherapy” OR “videotape” OR “audiotape” OR “etherapy” OR “cybertherapy” OR “e-health” OR “videoconferenc*” OR “videoteleconferenc*” “interapy” OR “tele*” OR “electronic” OR “skype” OR “instant messaging” OR “mobile” OR “tape” OR “DVD*” OR “CD*” OR “self-help” OR “self-care” OR “self-directed” OR “self-change” OR “self-management” OR “self-administ*” OR “guided self-help” OR “guided self-change” OR “guided” OR “self-exposure” OR “minimal contact” OR “minimal therapist contact” OR “reduced contact” OR “reduced therapist contact” OR “limited contact” OR “limited therapist contact” OR “therapist assisted”) [Topic]
3. (“random*” OR “factorial” OR “crossover*” OR “cross over*” OR “cross-over” OR “placebo” OR (doubl* blind*) OR (singl* blind*) OR “assign*” OR “allocate*” OR “volunteer*” OR “control*” OR (clinical trial)) [Topic]
4. 1 AND 2 AND 3

**Proquest Dissertation**

1. (“posttraumatic stress disorder” OR “post-traumatic stress disorder” OR “PTSD” OR “post traumatic stress disorder” OR “posttraumatic” OR “post-traumatic” OR “traumatic event”) [Title, Abstract]
2. (“web*” OR “comput*” OR “internet” OR “online” OR “bibliotherapy” OR “videotape” OR “audiotape” OR “etherapy” OR “cybertherapy” OR “e-health” OR “videoconferenc*” OR “videoteleconferenc*” “interapy” OR “tele*” OR “electronic” OR “skype” OR “instant messaging” OR “mobile” OR “tape” OR “DVD*” OR “CD*” OR “self-help” OR “self-care” OR “self-directed” OR “self-change” OR “self-management” OR “self-administ*” OR “guided self-help” OR “guided self-change” OR “guided” OR “self-exposure” OR “minimal contact” OR “minimal therapist contact” OR “reduced contact” OR “reduced therapist contact” OR “limited contact” OR “limited therapist contact” OR “therapist assisted”) [Title, Abstract]
3. (“random*” OR “factorial” OR “crossover*” OR “cross over*” OR “cross-over” OR “placebo” OR (doubl* blind*) OR (singl* blind*) OR “assign*” OR “allocate*” OR “volunteer*” OR “control*” OR (clinical trial)) [Title, Abstract]
4. 1 AND 2 AND 3

**OpenGrey**

“Posttraumatic stress disorder” OR “post-traumatic stress disorder” OR “PTSD” OR “post traumatic stress disorder” OR “posttraumatic” OR “post-traumatic” OR “traumatic event” [Abstract]

**List of all included papers**

1. *Acosta MC, Web-Delivered CBT reduces heavy drinking in OEF-OIF veterans in primary care with symptomatic substance use and PTSD. *Behavior Therapy* 2017; **48**: 262–276.
2. *Engel CC, Litz B, Magruder KM, Harper E, Gore K, Stein N, et al. Delivery of self training and education for stressful situations: A randomized trial of nurse assisted online self-management for PTSD in primary care. *Gen Hosp Psychiatry* 2015; **37:** 323–328.
3. *Gawlytta R, Kesselmeier M, Scherag S, Niemeyer H, Böttche M, Knaevelsrud C, et al. Internet-based cognitive-behavioral writing therapy for reducing posttraumatic stress after severe sepsis in patients and their spouses (REPAIR): Results of a randomized controlled trial. 2017.
4. *Ivarsson D, Blom M, Hesser H, Carlbring P, Enderby P, Nordberg R, Andersson G. Guided internet-delivered cognitive behavior therapy for post-traumatic stress disorder: A randomized controlled trial. *Internet Interv* 2014; **1**: 33-40.
5. *Knaevelsrud C, Maercker, A. Long-term effects of an internet-based treatment for posttraumatic stress. *Cogn Behav Ther* 2009; **39**: 72-77.
6. *Knaevelsrud C, Brand J, Lange A, Ruwaard J, Wagner B. Web-based psychotherapy for posttraumatic stress disorder in war-traumatized Arab patients: Randomized controlled trial. *J Med Internet Res* 2015; **17**: 27–40.
7. *Knaevelsrud C, Bottche M, Pietrzak RH, Freyberger HJ, Kuwert P. Efficacy and feasibility of a therapist-guided internet-based intervention for older persons with childhood traumatization: A randomized controlled trial. *Am J Geriatr Psychiatry* 2017; **25** 878-888.
8. * Lange A, Rietdijk D, Hudcovicova M, van de Ven J-P, Schrieken B, Emmelkamp PMG. Interapy: A controlled randomized trial of the standardized treatment of posttraumatic stress through the internet. *J Consult Clin Psychol* 2003; **71**: 901–909.
9. *Latif M, Husain I, Gul M, Naz S, Irfan M, Aslam F, et al. Culturally adapted trauma-focused CBT-based guided self-help (CatCBT GSH) for female victims of domestic violence in Pakistan: feasibility randomized controlled trial. *Behav Cogn Psychother* 2021; **49**: 50–61.
10. *Lehovat K, Millard SP, Thomas RM, Yantsides K, Upham M, Beckham B, et al. A randomized trial of an online, coach-assisted self-management PTSD intervention tailored for women veterans *J Consult Clin Psychol* 2021; **89**: 134-141.
11. *Lewis CE, Farewell D, Groves V, Kitchiner NJ, Roberts NP, Vick, T, et al. Internet-based guided self-help for posttraumatic stress disorder (PTSD): Randomized controlled trial. *Depress Anxiety* 2017; **34**: 555–565.
12. *Litz BT, Engel CC, Bryant RA, Papa A. A randomized, controlled proof-of-concept trial of an Internet-based, therapist-assisted self-management treatment for posttraumatic stress disorder. *Am Journal Psychiatry* 2007; **164**: 1676–1683.
13. *Nieminen K, Berg I, Frankenstein K, Viita L, Larsson K, Persson U, et al. Internet-provided cognitive behaviour therapy of posttraumatic stress symptoms following Childbirth: A randomized controlled trial. *Cogn Behav Ther* 2016; **45**: 287-306.
14. *Sloan DM, Marx BP, Bovin MJ, Feinstein BA, Gallagher MW. Written exposure as an intervention for PTSD: A randomized clinical trial with motor vehicle accident survivors. *Behav Res Ther* 2012; **50**: 627-635.
15. *Spence J, Titov N, Dear BF, Johnston L, Solley K, Lorian C, et al. Randomized controlled trial of Internet-delivered cognitive behavioral therapy for posttraumatic stress disorder. *Depress Anxiety* 2011; **28** :541-550.
16. *Vinke YD, Truijens F, van Polanen Petel E, van Emmerik AAP. The efficacy of a single imaginal exposure‐writing assignment in reducing posttraumatic stress and depressive symptoms: A pilot study. *J Clin Psychol* 2019; **75**:1519–1526.
17. *Wagner B, Knaevelsrud C, Maercker A. Internet-based cognitive-behavioral therapy for complicated grief: a randomized controlled trial. *Death Stud* 2006; **30**: 429-453.

|  | 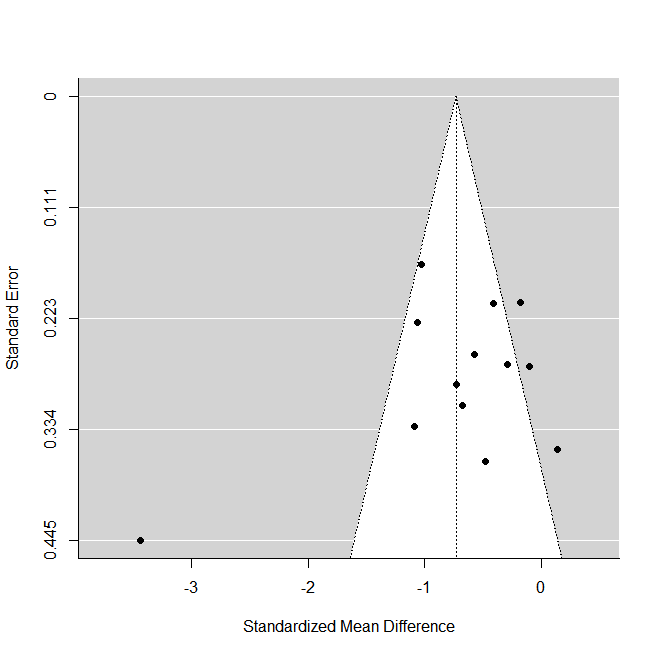 |
| --- | --- |
| PTSD | Depressive symptoms |
| 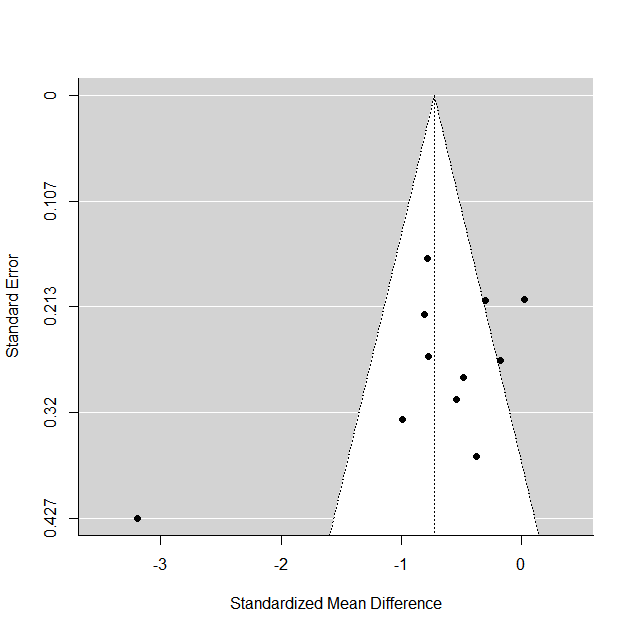 | |
| Anxiety symptoms | |

Fig S1. Funnel plots of effect sizes for PTSD, depressive symptoms, and anxiety symptoms.

|  |  |
| --- | --- |
| PTSD | Depressive symptoms |
|  | |
| Anxiety symptoms | |

Fig S2. Funnel plots of effect sizes for PTSD, depressive symptoms, and anxiety symptoms with one outlier removed from each funnel plot

**Publication bias**

Funnel plots were created to explore the distribution of effect sizes against their standard errors (see Fig S1 and S2). None of the funnel plots show a sparsely populated left side: the hallmark indicator of publication bias as a result of unpublished studies reporting small effect sizes or null-findings. Given that some degree of asymmetry is to be expected with relatively few data points (Sterne et al., 2011), the funnel plots generally appear fairly symmetrical and funnel-shaped, suggesting an absence of publication bias.

The Trim and Fill method further supported the absence of publication bias, identifying that 0 studies were missing to the right of the mean for each of the mental health conditions. The Test for Funnel Plot Asymmetry indicated potential publication bias for PTSD (*b* = 0.66, z = -2.08, *p* = 0.04) and anxiety symptom (*b* = 0.99, z = -2.35, *p* = 0.01) effect sizes, but not depressive symptom effect sizes (*b* = 0.66, z = -1.74, *p* = 0.08).

As discussed in the manuscript, some outliers were identified and these somewhat inflated effect size estimates. Removing the identified outliers nullified the potential publication bias identified by the Test for Funnel Plot Asymmetry for PTSD (*b* = -0.30, z = -0.53, *p* = 0.60), anxiety symptom (*b* = -0.41, z = 0.20, *p* = 0.84), and depressive (*b* = -0.97, z = 0.81, *p* = 0.41) symptom effect sizes.

Table S2. Risk of bias for included studies.

|  | Random sequence generation | Allocation concealment | Blinding of participants and personnel | Blinding of outcome assessment | Incomplete outcome data | Selective reporting | Other bias | Total score |
| --- | --- | --- | --- | --- | --- | --- | --- | --- |
| Acosta 2017 | Low risk | Unclear risk | High risk | Unclear risk | Low risk | Low risk | Low risk | 4 |
| Engel 2015 | Low risk | Unclear risk | High risk | Low risk | Low risk | Unclear risk | Low risk | 4 |
| Gawlytta in Press | Low risk | Unclear risk | High risk | Unclear risk | Unclear risk | Unclear risk | Unclear risk | 7 |
| Ivarsson 2014 | Low risk | Low risk | High risk | Low risk | Unclear risk | Unclear risk | Low risk | 4 |
| Knaevelsrud 2007 | Low risk | Unclear risk | High risk | Low risk | Unclear risk | Unclear risk | Low risk | 5 |
| Knaevelsrud 2015 | Low risk | Unclear risk | High risk | Low risk | Low risk | Unclear risk | Low risk | 4 |
| Knaevelsrud 2017 | Low risk | Low risk | High risk | Unclear risk | Unclear risk | Low risk | Low risk | 4 |
| Lange 2003 | Low risk | Unclear risk | High risk | Low risk | Low risk | Unclear risk | Low risk | 4 |
| Latif 2021 | Low risk | Low risk | High risk | High risk | Unclear risk | Unclear risk | Low risk | 6 |
| Lehovat 2021 | Low risk | Low risk | High risk | Unclear risk | Unclear risk | Low risk | Low risk | 4 |
| Lewis 2017 | Low risk | Low risk | High risk | Low risk | Low risk | Unclear risk | Low risk | 3 |
| Litz 2007 | Low risk | Unclear risk | High risk | Low risk | Unclear risk | High risk | Low risk | 6 |
| Nieminen 2016 | Low risk | Low risk | High risk | High risk | Low risk | Unclear risk | Low risk | 5 |
| Sloan 2012 | Low risk | Low risk | High risk | Low risk | Low risk | High risk | Low risk | 4 |
| Spence 2011 | Low risk | Low risk | High risk | Low risk | Unclear risk | Unclear risk | Low risk | 4 |
| Vinke 2019 | Low risk | Low risk | High risk | Low risk | Unclear risk | Unclear risk | Low risk | 4 |
| Wagner 2006 | Low risk | Unclear risk | High risk | Low risk | Unclear risk | Unclear risk | Low risk | 5 |
